# Comparing online and face-to-face administration of a neuropsychological computerized attention test: Assessment modality does not influence performance

**DOI:** 10.3389/fpsyg.2023.1134047

**Published:** 2023-04-26

**Authors:** Daniel Negrini, Sergio L. Schmidt

**Affiliations:** ^1^Department of Anesthesiology, Federal University of the State of Rio de Janeiro, Rio de Janeiro, Brazil; ^2^School of Medicine, Fluminense Federal University, Niterói, Brazil; ^3^Department of Neurology, Federal University of the State of Rio de Janeiro, Rio de Janeiro, Brazil

**Keywords:** attention (AT), face-to-face, culture, retest, teleneuropsychology

## Abstract

**Background:**

The cognitive impairment associated with the COVID-19 pandemic highlighted the need for teleneuropsychology (1). Moreover, neurologic diseases associated with mental deterioration usually require the use of the same neuropsychological instrument to assess cognitive changes across time. Therefore, in such cases, a learning effect upon retesting is not desired. Attention and its subdomains can be measured using Go/no-go tests, such as, the Continuous Visual Attention Test (CVAT). Here, we administered the CVAT to investigate the effect of modality (online vs. face-to-face) on attentional performance. The variables of the CVAT measures four attention domains: focused-attention, behavioral-inhibition, intrinsic-alertness (reaction time, RT), and sustained-attention (intra-individual variability of RTs, VRT).

**Methods:**

The CVAT was applied face-to face and online in 130 adult Americans and 50 adult Brazilians. Three different study designs were used: (1) Between-subjects design: healthy Americans were tested face-to-face (*n* = 88) or online (*n* = 42). We verified if there were any differences between the two modalities. (2) Within-subjects design: Brazilians participants (*n* = 50) were tested twice (online and face-to-face). For each CVAT variable, repeated measures ANCOVAs were performed to verify whether modality or first vs. second tests differ. Agreement was analyzed using Kappa, intraclass correlation coefficients, and Bland–Altman plots. (3) Paired comparisons: we compared Americans vs. Brazilians, pairing subjects by age, sex, and level of education, grouping by modality.

**Results:**

Assessment modality did not influence performance using two independent samples (between-subjects design) or the same individual tested twice (within-subjects design). The second test and the first test did not differ. Data indicated significant agreements for the VRT variable. Based on paired samples, Americans did not differ from Brazilians and a significant agreement was found for the VRT variable.

**Conclusion:**

The CVAT can be administered online or face-to-face without learning upon retesting. The data on agreement (online vs. face-to-face, test vs. retest, Americans vs. Brazilians) indicate that VRT is the most reliable variable.

**Limitations:**

High educational level of the participants and absence of a perfect balanced within-subjects design.

## 1. Introduction

There has been an increasing interest in online cognitive testing (Marra et al., 2020). Particularly, the COVID-19 pandemic affecting a great number of individuals worldwide, highlighted the need to answer what could be the similarities or differences between online vs. face-to-face assessments (Gates and Kochan, 2015; Sumpter et al., 2023), as well as differences across cultural boundaries. Moreover, long COVID and several other diseases have been found to be associated with progressive cognitive changes. Therefore, the effect of retesting is also a topic of interest (Webb et al., 2022).

The importance of reliability between test and retest is a topic of interest since we need to be sure that eventual fluctuations across time do have clinical significance, instead of being consequence of any learning effect. Also, the use of teleneuropsychology faces many practical obstacles and requires comparisons with face-to-face evaluations (Rochette et al., 2022). Besides the relevance of teleneuropychology and test vs. retest reliability, there is a lack of studies on these issues. As attention is considered pivotal to the proper function for all other cognitive domains (Lezak, 1983; Balsimelli et al., 2007), the present study focused on the attention subdomains to investigate whether threre were differences between online and face-to-face modalities, and bewteen the first and the second test.

The Continuous Visual Attention Test (CVAT) is Go/no-go task (Schmidt et al., 2020) that has been frequently used to assess the attention subdomains in several neurologic diseases (Simões et al., 2018; Schmidt et al., 2021). The number of omission errors (OE) reflects problems on focused attention, whereas the number of incorrect hits or false alarms (no-go) indicates response inhibition, i.e., commission errors (CE; Sessler et al., 2002). Intrinsic alertness is assessed by measuring average visuomotor reaction times (RT) for the correct hits (Simões et al., 2018). Sustained attention is assessed considering the intraindividual variability of RTs (VRT), which measures the fluctuation in RTs across the test (Simões et al., 2018). The short version of the CVAT takes only 90 s to complete. It has been proven to provide useful information on cognition in different clinical scenarios. In elderly patients with mild cognitive impairment and early Alzheimer’s disease, the VRT variable of the short version of the CVAT has been shown to progressively worsen with increasing level of cognitive impairment (Schmidt et al., 2020). Also, individuals recovering from COVID-19 present worsened performance on some variables of the short version of the CVAT (Do Carmo Filho et al., 2022; Schmidt et al., 2023).

The present work has four aims. (1) To verify if the test modality (online vs. face-to-face) affected CVAT’s performance using the short version of the CVAT in different individuals, one group submitted to online and the other group to face-to-face testing (between-subjects study design); (2) To assess if the test modality (online vs. face-to-face) affected CVAT’s performance using the same individual tested in both modalities (within-subjects study design); (3) To test if there was a learning effect upon retest in the same subject (within-subjects study design); and (4) To compare CVAT’s performance between Americans and Brazilians (paired comparisons matched by age, sex, and educational level).

## 2. Materials and methods

### 2.1. Participants

#### 2.1.1. *A priori* calculation of the minimum sample size for each study design (power analysis and sample size)

To estimate the required sample size, we performed a power analysis. As we used two different study designs (within- and between-subjects), different analyses were performed. For both conditions, α = Type I error = 0.05 and β = Type II error = 0.20 (power = 1–β = 0.80) were applied. Here, we performed MANCOVAs because they would account for any potential correlations between the dependent variables. Thus, hypothetically, the MANCOVAs could show significant differences between the means while the individual ANCOVAs and the *t*-tests did not. However, irrespective of the results of the MANCOVAs, we always performed *post hoc* tests to determine where there were significant differences (i.e., which specific independent variable level significantly differs from another). As all the *post hoc* comparisons were variations of t tests, we performed power analyses considering *t*-tests in the two different designs (independent and paired *t*-tests, respectively).

For the between-subjects design, we estimated the minimum differences (Δ) considering that they must reach magnitude levels that have clinical significance. For each variable of the test, the population standard deviation (σ) and the mean difference (Δ) with a real clinical significance were estimated based on comparisons (larger samples in previous studies) between healthy controls and patients with clinically defined attention disorders. We considered relevant: Δ_OE_ = 4 errors; Δ_CE_ = 6 errors; Δ_RT_ = 60 ms, and Δ_VRT_ = 20 ms. Then, we found the following values for Cohen’s d: d_OE_ = 1.1; d_CE_ = 1.5; d_RT_ = 1.2; and d_VRT_ = 0.87. Since the expected difference could be small or none, we selected the smallest clinically relevant effect size. Accordingly, we performed power analysis with the lowest Cohen’s d among the four CVAT variables, i.e., 0.87. For an allocation ratio of 2, we found the following sample sizes: *n*_group1_ = 13 and *n*_group2_ = 27.

For the within-subjects design, we also estimated the minimum differences (Δ) considering that they must reach magnitude levels that have clinical significance. The σ and the mean differences with a real clinical significance were based on comparisons involving average differences between patients with clinically defined attention disorders and age- and sex-matched paired controls. Although the values of the Δ_s_ remained unchanged, the values of σ_s_ were smaller compared to the between-subjects design. As described for the between-subjects design, we performed power analysis considering the smallest Cohen’s d, i.e., 1. We found a sample size of 10 subjects (Akens, 2013).

Considering the minimum sample size, we recruited at least three times more subjects for each design.

#### 2.1.2. Exclusion criteria

The general exclusion criteria for the two groups were as follows: age > 50 or < 18 years; taking antipsychotic, anti-seizure, or any medication that could interfere with attention performance; reduced kidney or hepatic function; past head trauma and loss of consciousness; current alcohol/substance use disorder; pre-existing neurologic or psychiatric disorders; non-corrected hearing or visual impairments; and previous cognitive impairment.

#### 2.1.3. Between-subjects study design (American participants)

Considering the exclusion criteria, we succeeded analyzing a total of 130 health Americans (88 face-to-face and 42 online) between September 14, 2019 and June 12, 2020. They were recruited from the division of surgical oncology at the University of Colorado (United States), Anschutz Medical Campus.

For the face-to face modality (*n* = 88), the CVAT was administered on a quiet and silent room at the office building at the Anschutz Medical Campus (University of Colorado—United States), under supervision of a research staff.

For the online modality (*n* = 42), the CVAT software was sent by email to be completed at home and sent back to a research staff. When this was the case, specific instructions on how to proceed with the test were given in clear and concise language.

Data regarding age, gender, handedness, educational level, and ethnicity (Afro Americans vs. Non-Afro Americans) were also collected.

The demographic characteristics of the American sample, grouped by online vs. face-to-face, are shown in Table 1. There were no statistically significant differences between the two subgroups, for any of the demographic variables collected.

**Table 1:** Demographic characteristics of American participants according to the test modality (online vs. face-to-face).

	Online (*n* = 42)	Face-to-face (*n* = 88)	Total (*n* = 130)	*p* value
Age	36.48 (9.33)	35.51 (9.24)	35.82 (9.24)	0.58
Sex				0.97
Female	18 (42%)	38 (43%)	56 (43%)	
Male	24 (58%)	50 (57%)	74(57%)	
Educational level				0.09
Undergraduate	4 (9%)	19 (21%)	23 (17%)	
Graduate	25 (59%)	36 (40%)	61 (46%)	
Post-graduate	13 (32%)	33 (39%)	46 (37%)	
Handedness				0.78
Left	5 (11%)	12 (13%)	17 (13%)	
Right	37 (89%)	76 (87%)	113 (87%)	
Ethinicity				0.39
Afro	21 (50%)	37 (42%)	58 (44%)	
Non-Afro	21 (50%)	51 (58%)	72 (56%)	

Continuous variables are presented as mean (SD). Categorical variables are presented as frequency (percentage). SD, standard deviation; Age in years.

#### 2.1.4. Within-subject study design (Brazilian participants)

We collected data from 50 Brazilian medical students between August 2nd and August 09th 2022. They were recruited from two Federal Universities at the State of Rio de Janeiro (Brazil).

Twenty-six participants performed the CVAT face-to-face, under similar conditions to the American sample, while 24 subjects were tested online. One week later, the same subjects that had been tested face-to-face were tested online, while the other part, previously tested online, were assessed face-to-face.

Regarding the Brazilian sample, the comparisons between those who tested first face-to-face to those who tested first online are shown in Table 2. There were no statistically significant demographic differences between these two subgroups.

**Table 2:** Demographics characteristics of Brazilian participants according to the sequence of testing (sequence 1 = face-to-face 1st; sequence 2 = online 1st).

	Sequence 1 (*n* = 26)	Sequence 2 (*n* = 24)	Total (*n* = 50)	*p* value
Age	24.61 (5.76)	25.25 (4.43)	24.92 (5.13)	0.67
Sex				0.56
Female	13 (50%)	14 (58%)	27 (54%)	
Male	13 (50%)	10 (42%)	23 (46%)	

Continuous variables are presented as mean (SD). Categorical variables are presented as frequency (percentage). SD, standard deviation; Sequence 1, first test face-to-face; Sequence 2, first test online; and age in years.

#### 2.1.5. Paired comparisons (Americans vs. Brazilians)

For the paired comparisons, the level of education of the American sample was classified into Undergraduate, Graduate, and Post-Graduate. In the Brazilian sample, all the participants were medical students. Considering age, sex and educational level, 15 pairs were analyzed face-to-face and 10 pairs online.

For the paired comparisons, the mean age and respective standard deviation (SD) in years, for those tested online, were 30.86 (6.88). For those tested face-to-face, were 30.06 (7.50), respectively.

### 2.2. Procedures

**Figure 1:**
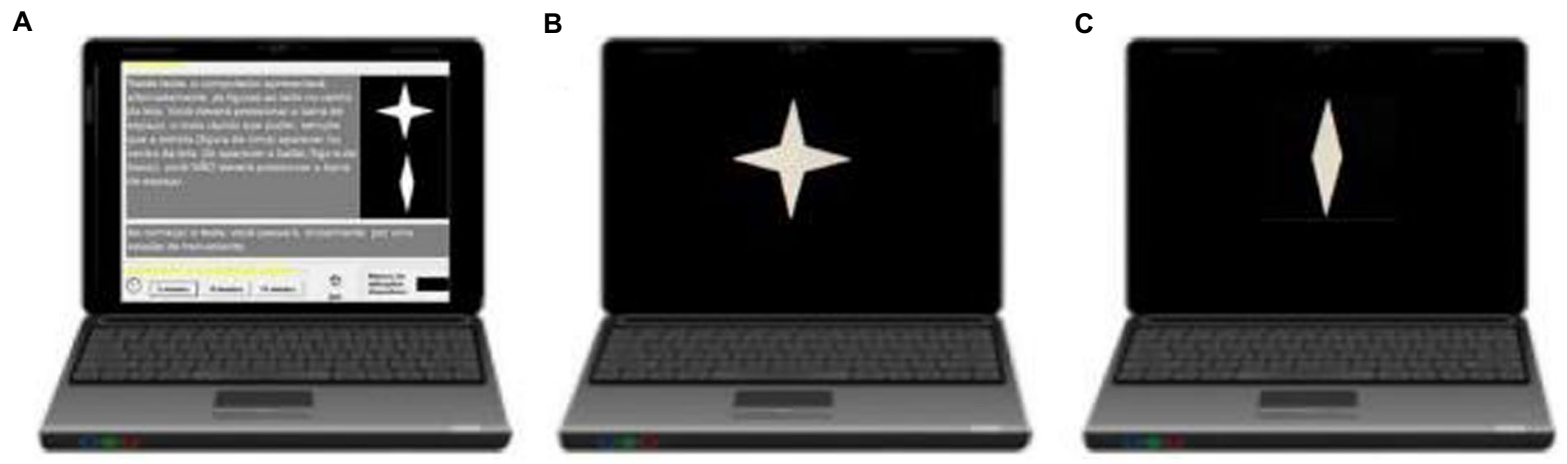
Computer visual attention test (CVAT). The test begins with written instructions on the screen **(A)**. The target **(B)** remains on the screen for 250 ms. The non-target **(C)** remains on the screen for 250 ms. Inter-stimulus time interval is 1 s. The test lasts 90 s. Instructions in English goes as follows: “In this test, the computer alternately displays the indicated figures in the center of the screen. You must press the spacebar using your dominant hand as fast as you can whenever the star appears in the center of the screen. If the other figure appears, you should not press the space bar.” Adapted from Schmidt et al. (2019).

The CVAT (Figure 1) was administered to all participants. Subjects taking the test face-to-face were instructed to sit in front of a computer. The distance between the center of the monitor and the eyes was approximately 50 cm. The examiner instructed the subject, either face-to-face or by instruction *via* email, to press the spacebar on the keyboard as fast as possible each time a specific target was displayed. The test started with instructions and a practice session. The practice sessions took 10 s. A second practice session was administered if the participant failed the first one. Only participants who succeeded in the practice session (first or second) were allowed to continue the experiment. The main task consisted of 90 trials (two figures presented, one each time, target, or non-target), 72 correct targets and 18 non-targets. The inter-stimulus time interval was 1 s. Each stimulus was displayed for 250 ms. The test took 1.5 min to complete. The types of measures included omission errors (OE, focused attention), commission errors (CE, response inhibition), average reaction time of correct responses (RT, intrinsic alertness), and variability of correct reaction times (VRT, sustained attention). VRT was estimated by a per-person measure of the standard deviation (SD) of individual RTs for the correctly signaled targets. Previous studies have shown that RT and VRT can be reliably measured by tests as short as 52 s with 20 items (Manuel et al., 2019). Subjects assigned to perform the test online were specifically instructed by e-mail, in clear and concise language, on how to proceed with the test.

### 2.3. Statistical analysis

All the statistical analyses were conducted with the SPSS version 26, considering a value of *p* <5% (two tailed) as significant.

The results from the Brazilian participants were compared to the values of a reference group, stratified according to age and sex. This reference group consisted of healthy subjects taking a mandatory medical and psychological exam for a certificate of fitness to drive, who voluntarily performed the short version of the CVAT. This subsample has been previously described by Do Carmo Filho et al. (2022). To proceed with the analysis, we calculated the percentiles for each participant based on the frequency distributions for each CVAT variable from the reference values. The frequency distributions were stratified according to five age ranges (20–29, 30–39, 40–49, 50–59, and 60–69). For each CVAT variable, a participant’s performance equal to or above the value of the 90th percentile was classified as being significantly impaired. For instance, a RT of 400 ms for an individual with 29 years old is above the 90th percentile, compared to the reference value.

#### 2.3.1. Between-subjects design

To verify whether there was significant differences among participants tested only online or face-to-face, a MANCOVA was performed including RT, VRT, OE, and CE as dependent variables, and the modality of testing (online vs. face-to-face) as the independent variable. Age, sex, educational level, handedness, and ethnicity (Afro vs. Non-Afro Americans) were used as co-variates. Box’s M-test was used to assess the homogeneity of the covariance matrices. A significant MANCOVA indicates that at least one dependent variable is different between the groups, thus allowing for further *post hoc* univariate ANCOVAs. A MANCOVA/ANCOVA approach was chosen as it has been shown to give robust results even when variables are not normally distributed (Algina and Olejnik, 1984). To find out whether there were mean differences between the two modalities, we also performed independent *t*-tests on the CVAT variables.

As part of a robust data analysis, we used the Wald-Wolfowitz test and the Kolmogorov–Smirnov test to verify differences between online and face-to-face distributions for each parameter of the CVAT. These two tests verified where the distributions (online vs. face-to-face) differed in means, variances, or shapes. Kolmogorov–Smirnov test has been shown to be more powerful than the Wald-Wolfowitz test for detecting differences solely in their location whereas the Wald-Wolfowitz is more powerful if the distributions differ in solely in variance and have small differences in locations (Magel and Wibowo, 1997).

To verify whether there were statistically significant differences between the medians of online and face-to-face CVAT data, we used the Kruskal-Wallis-Test (Kruskal and Wallis, 1952). We also applied the Jonckheere-Terpstra test (Jonckheere, 1954) whether online and face-to-face samples were from the same population. We applied the Moses test of extreme reaction (Moses, 1952) to test whether extreme values are equally likely in online and face-to-face populations or if they are more likely to occur in the population from which the sample with the larger range was drawn.

Additionally, box plots were used to compare the distribution of data between online and face-to-face from different individuals.

Even though the between-subjects comparisons make the analysis less simple, it has the advantage of avoiding any potential learning bias effect.

#### 2.3.2. Within-subjects design

In this design, the same subject was exposed to both modalities (online and face-to-face) at two different moments (test and retest). Sequence of testing across the two modalities was counterbalanced: 26 participants started with face-to-face, and 24 with online testing. The analysis included: comparisons of mean differences, agreements based on categorical variables and continuous variables, and correlation analysis for each variable of the CVAT.

Means comparisons were performed using repeated-measures MANCOVAs using the following covariates: sex, age, and the two sequences of testing modality (sequence 1: first test face-to-face followed by online; sequence 2: first online followed by face-to-face). Within-subject factor: modalities (online and face-to-face), or time (test and retest). Following the MANCOVAs, respective ANCOVAs and paired *t*-tests were also performed.

We tested the agreement between modalities, as well as between test and retest, considering two excluding dichotomic categories. Subjects were considered normal when performing up to the percentile 90th, compared to the reference values, and non-normal when performing above percentile 90th. The Kappa Statistic (Cohen’s* Kappa) was calculated to measure agreement between the two modalities and between the first and the second test.

Intraclass correlation (ICC) was used to estimate inter-modality and test–retest agreement on raw data derived from the CVAT variables. We used the two-way mixed model because we assumed a random effect of the CVAT data and a fixed effect of the two modalities.

Bland–Altman plots (B-A) were also used to assess agreement between measurements on the same subject. For the B-A analysis, a scatter plot was constructed in which the difference between the paired measurements was plotted on *y*-axis and average of both measurements on *x*-axis. The bias (mean difference in values obtained with both measurements) was represented by a central horizontal line on the plot. The standard deviation (SD) of the differences between paired measurements was used to construct horizontal lines above and below the central horizontal line to represent 95% limits of agreement (LOA; mean bias ±1.96 SD). Conclusions on agreement and interchangeability of both measurements were made based upon the width of these LOA in comparison to *a priori* defined clinical criteria as defined in the power analysis. For each CVAT variable, we checked if there was heteroscedastic distribution (i.e., whether the magnitude of differences increases proportionally to the size of the measurement).

Finally, a correlation analysis was conducted to verify the relationship between the attentional performance in the two modalities, as well as test and retest. For each CVA variable, the Pearson product moment correlation coefficient was calculated.

#### 2.3.3. Paired comparisons

For each test modality, online and in face-to-face, Americans and Brazilians were paired and matched by age, educational level, and sex. The pairs were grouped by modality of testing. Paired *t*-tests were performed to find out whether there were significant mean differences in the CVAT performance between Americans and Brazilians. ICC was used to estimate raw data agreements between Brazilians and Americans.

### 2.4. Ethics aspects

The participation in the study was voluntary, and the research protocol was declared exempted by the local ethical committee (COMIRB: 20-0423) from the University of Colorado (United States), Anschutz Medical Campus. Also, it was approved by the ethical committee of the Federal University of the State of Rio de Janeiro (Brazil; CAE: 61259922.4.0000.5258). The study was performed in accordance with the Helsinki Declaration. Informed written consent was obtained from the participants.

## 3. Results

### 3.1. Comparisons between two modalities of testing using different individuals (between-subjects design)

The descriptive results of the four dependent variables of the CVAT, for online vs. face-to-face testing, are shown in Table 3. The overall MANCOVA did not reach statistical significance, *F* (4, 130) = 2.05, *p* = 0.94. Furthermore, subsequent ANCOVAs and independent *t*-tests did show any significant differences.

**Table 3:** Descriptive results of the CVAT for the American participants according to the testing modality (online vs. face-to-face).

CVAT variables	Mean	SD	*N*
CE			
Online	4.76	2.54	88
Face-to-face	4.33	2.41	42
Total	4.62	2.50	130
OE			
Online	0.40	0.85	88
Face-to-face	0.67	0.87	42
Total	0.48	0.87	130
RT			
Online	376.17	26.50	88
Face-to-face	376.69	26.16	42
Total	376.34	26.29	130
VRT			
Online	76.30	18.59	88
Face-to-face	76.00	17.88	42
Total	76.20	18.29	130

CE, commission errors (in units); OE, omission errors (in units); RT, correct reaction time (in ms); VRT, variability of correct reaction time (in ms); and SD, standard deviation.

**Figure 2:**
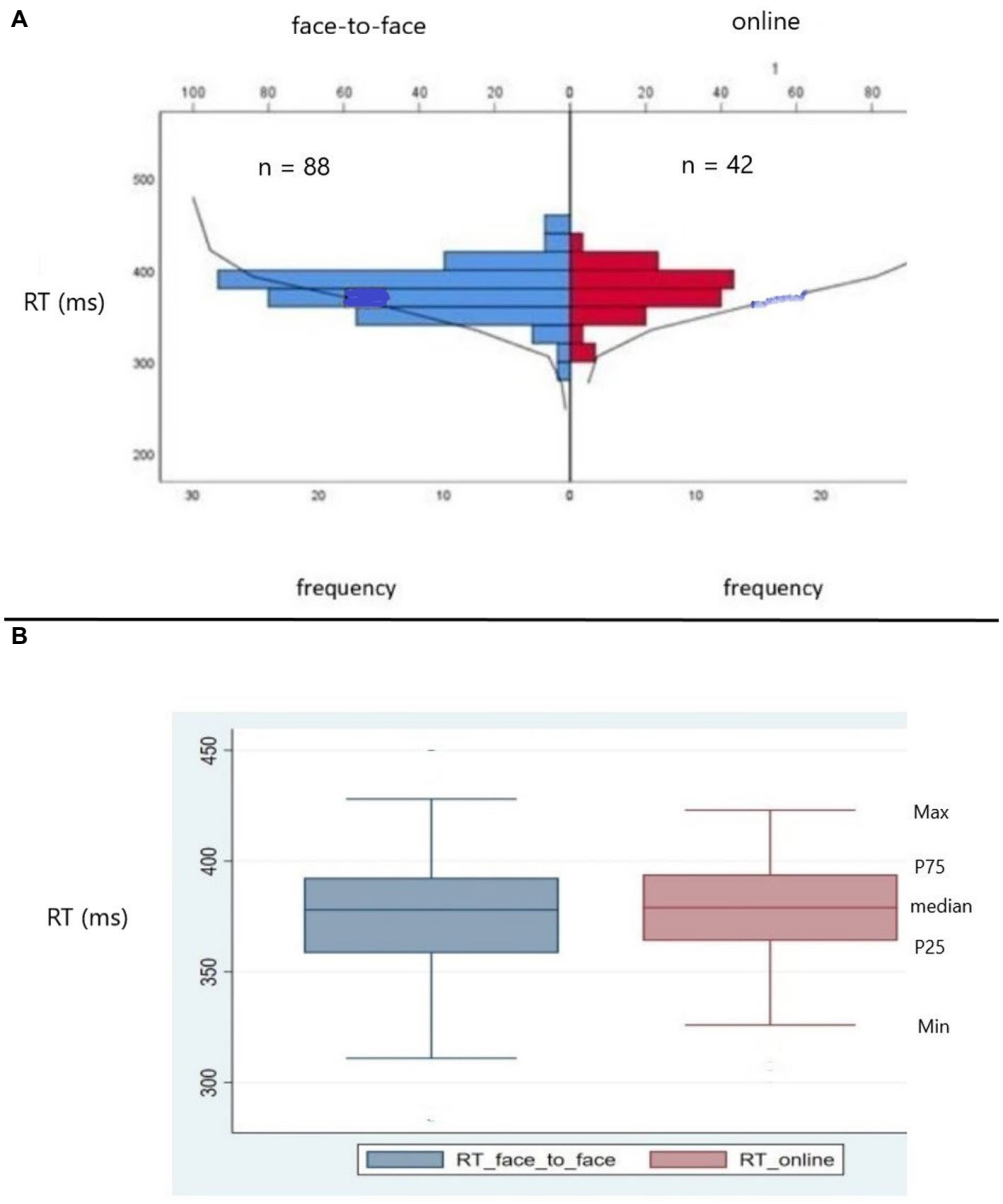
**(A)** Kolmogorov–Smirnov test (KS) for independent samples for correct reaction time (RT) comparing the distributions of data from American participants tested online and face-to-face In red RT data from participants exposed to online test; in blue, RT data from participants exposed to face-to-face test. The frequency distributions of RT for the two modalities did not differ (KS = 0.48, *p* > 0.70, two sides, ns). ns = non-significant. **(B)** Box plots showing median, percentile 25th (P25), percentile 75th (P75), minimum, and maximum RT values. In red, RT data from participants exposed to online test. In blue, RT data from participants exposed to face-to-face test. Observe that the frequency distributions for both modalities are similar.

**Figure 3:**
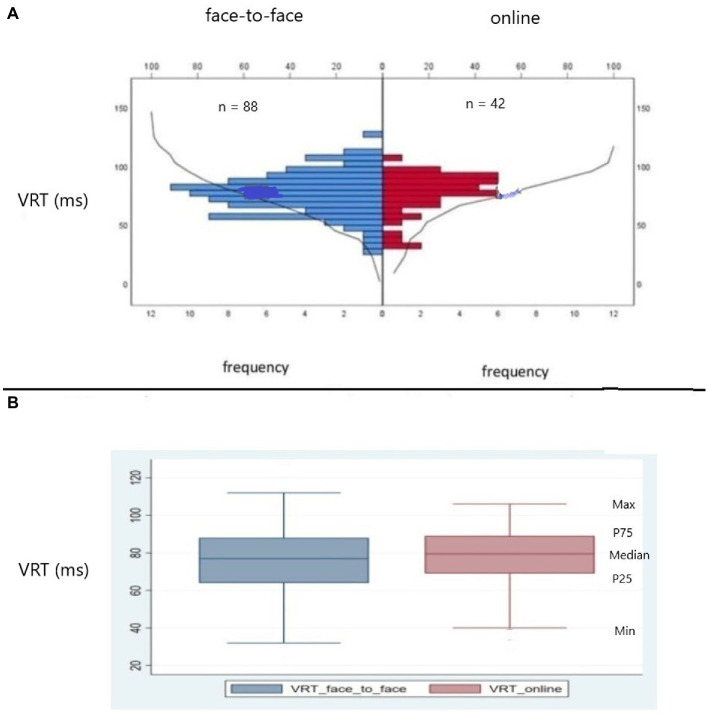
**(A)** Kolmogorov–Smirnov test (KS) for independent samples for variability of correct reaction time (VRT) comparing the distributions of data from American participants tested online and face-to-face. In red VRT data from participants exposed to online test; in blue, VRT data from participants exposed to face-to-face test. The frequency distributions of VRT for online and face-to-face modalities did not differ (KS = 0.59, *p* > 0.70, two sides, ns). ns = non-significant. **(B)** Box plots showing median, percentile 25th (P25), percentile 75th (P75), minimum, and maximum VRT values. In red, VRT data from participants exposed to online test. In blue, VRT data from participants exposed to face-to-face test. Observe that the frequency distributions for both modalities are similar.

As part of a robust statistical analysis, we conducted a series of tests to verify the distribution of data between online and face-to-face modalities. We figured out that there were no significant differences between the modality of testing (online vs. face-to-face), except for the extreme values and the medians of the OE variable. In fact, OE failed to keep the null hypothesis in the following tests: Moses, Mann–Whitney, Kruskal-Wallis, and Jonckeheere-Terpstra. The complete test of hypothesis is shown in Table 4. Visual demonstration of the data distribution and the Kolmogorov–Smirnov test for independent samples for RT and VRT, as well as the respective box plots, for both online vs. face-to-face, are shown in Figures 2A,B, 3A,B.

**Table 4:** Series of tests of hypothesis for data regarding the comparisons between modalities (online vs. face-to-face) using different individuals (between-subjects design).

Test of Hypothesis → CVAT Variables ↓	Wald-Wolfowitz	Moses	Mann–Whitney	Kolmogorov–Smirnov	Kruskal–Wallis-Test	Jonckheere-Terpstra
OE	NS	*	*	NS	*	*
CE	NS	NS	NS	NS	NS	NS
RT	NS	NS	NS	NS	NS	NS
VRT	NS	NS	NS	NS	NS	NS

OE, omission errors; CE, commission errors; RT, correct reaction time; VRT, variability of correct reaction time; ^*^p < 0.05; NS, non-significant.

### 3.2. Comparisons between two modalities using the same individuals (within subjects design)

The modality of the test did not influence the performance. The overall MANCOVA did not reach statistical significance, *F* (4, 43) = 0.66, *p* = 0.62. Subsequent ANCOVAs and paired *t*- tests did not indicate any significant difference between the two modalities.

We tested the percentage of clinical agreement between both modalities with the Kappa agreement analysis. Results for each dependent variables of the CVAT are shown in Table 5 and reached statistical significance for agreement on VRT (Kappa: 0.32, *p* = 0.03, percentage of agreement = 88.60%).

**Table 5:** Percentage of clinical agreement between modalities (online vs. face-to-face) for the Brazilian participants, with the Kappa agreement analysis.

	Kappa value	*p* value	Percentage of agreement
VRT	0.323	0.032	88.60%
RT	−0.023	0.877	97.70%
OE	0.033	0.826	64%
CE	0.136	0.328	75%

CE, commission errors; OE, omission errors; RT, correct reaction time; and VRT, variability of correct reaction time.

The ICC achieved significance for VRT (ICC = 0.53, df = 43, *p* = 0.01). In contrast, for the other variables of the CVAT, the ICC did not reach statistical significance.

**Figure 4:**
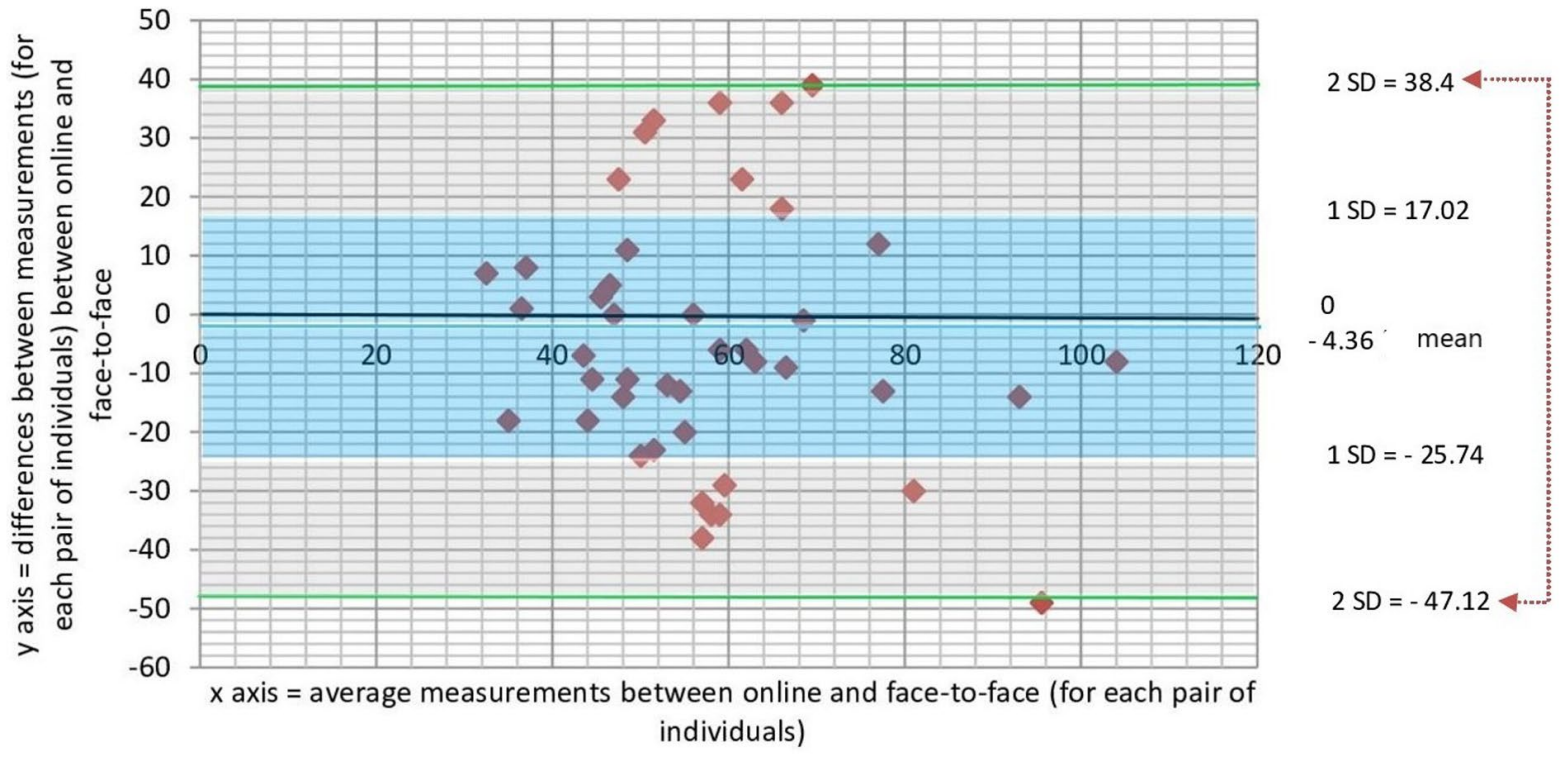
Scatter plot showing the relationship between magnitude of difference and size of measurement for the VRT variable measured online and face to face in the same subject. The difference between the paired measurements is plotted on the vertical-axis and average of the measures of two modalities (online and face-to-face) on the horizontal-axis. The mean difference in values obtained with the two modalities (bias) is represented by a central horizontal line on the plot. Horizontal lines above and below the central horizontal line represent the 95% limits of agreement (LOA—upper and lower LOA). *A priori* defined clinical criteria considered clinically relevant differences those that are greater than 20 ms. Note that all values are between ±2 SD from the mean, and more than 70% of values are within ±1SD from the mean. VRT, variability of reaction time.

Bland–Altman plot for values of VRT in the modalities online and face-to-face are shown in Figure 4. Values were found to be distributed around the mean value for the differences, and inside the 95% confidence interval. Considering the clinical criteria (Δ = 20 ms), more than 70% of the differences between measurements were not outside the clinically pre-defined relevant limits. Taking together, these findings strengthened the agreement for VRT between online and face-to-face measurements on the same subject.

**Figure 5:**
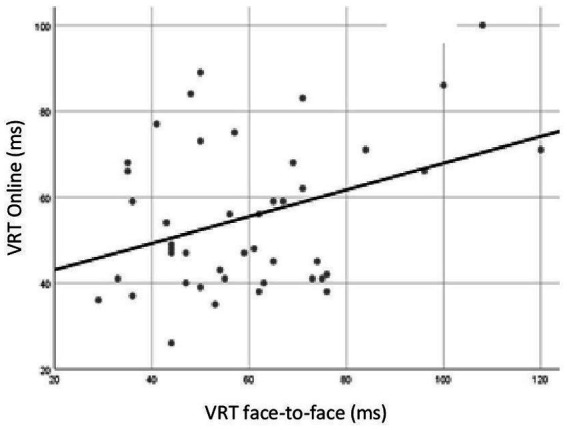
Pearson correlation analysis for variability of correct reaction time (VRT) of Brazilians participants tested online and face-to-face. In the horizontal axis, VRT data for participants exposed to face-to face test. In the vertical axis, VRT data for participants exposed to online test. Correlation was statistically significant (*r* = 0.36, *p* = 0.02).

Pearson correlation coefficient reached statistical significance for the VRT, as shown in Figure 5 (*r* = 0.36, *p* = 0.02). For the other variables, the coefficients did not reach significance.

### 3.3. Evaluation of learning effect (within-subjects design)

Test and retest showed no statistically significant differences in any of the four dependent variables of the CVAT. The overall MANCOVA did not reach statistical significance, *F* (4, 43) = 0.17, *p* = 0.68. Accordingly, subsequent ANCOVAS and paired *t*-tests did not indicate any significant difference between the first and the second tests.

We also tested the percentage of clinical agreement between test vs. retest with the Kappa agreement analysis. Results for each dependent variable of CVAT are shown in Table 6 and reached statistical significance for VRT (Kappa = 0.28, *p* = 0.05, percentage of agreement = 90.9%).

**Table 6:** Percentage of clinical agreement between testing across time (test vs. retest) for the Brazilian participants, with the Kappa agreement analysis.

	Kappa value	*p* value	Percentage of agreement
VRT	0.28	0.05	90.90%
RT	0	-	100.00%
OE	0.041	0.78	79.50%
CE	0.083	0.58	81.80%

CE, commission errors; OE, omission errors; RT, correct reaction time; VRT, variability of correct reaction time.

The ICC achieved significance for VRT (ICC = 0.54, df = 43, *p* = 0.01). In contrast, the ICC for the other CVAT’s dependent variables did not reach statistical significance.

**Figure 6:**
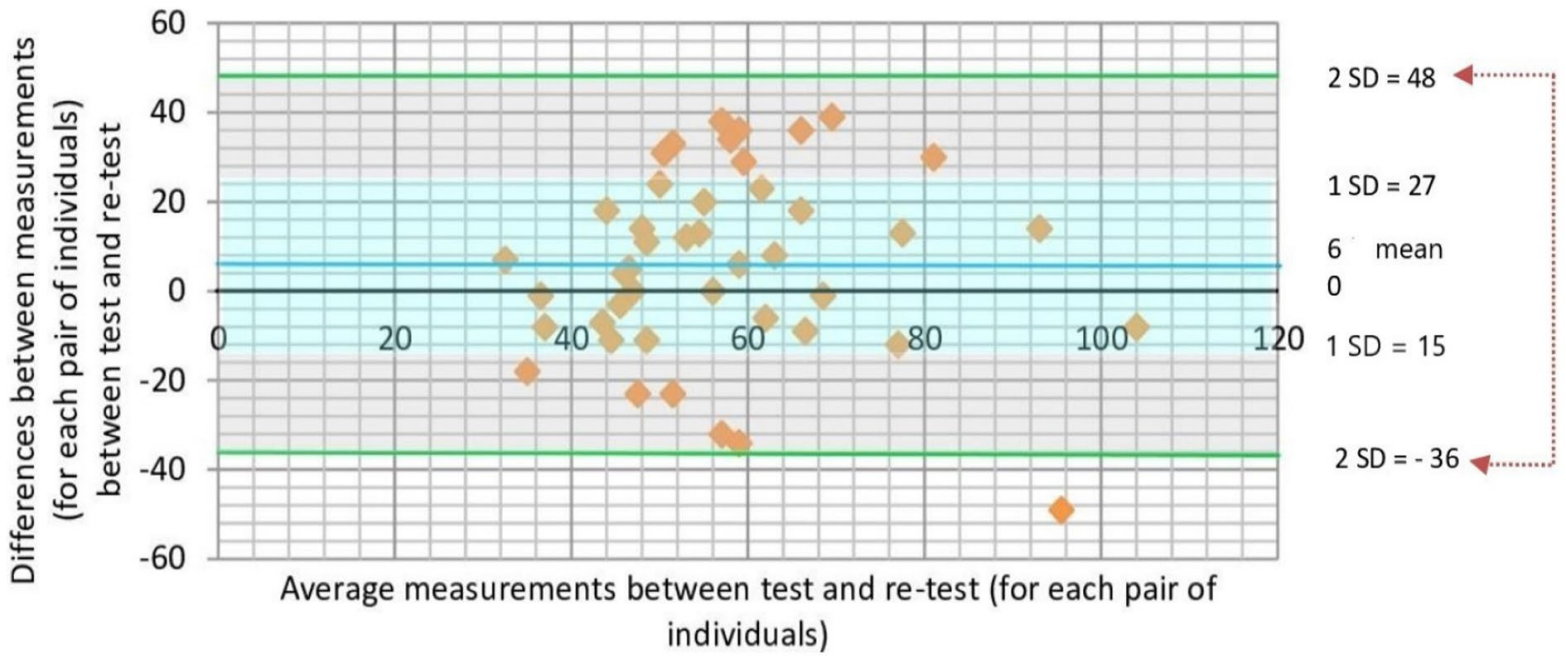
Scatter plot showing the magnitude of difference and size of measurement for the VRT variable measured in the first and second tests using the same subject. The difference between the paired measurements is plotted on the vertical-axis and average of the measures of first and second tests on the horizontal-axis. The mean difference in values obtained with test and retest (bias) is represented by a central horizontal line on the plot. Horizontal lines above and below the central horizontal line represent the 95% limits of agreement (LOA—upper and lower LOA). *A priori* defined clinical criteria considered clinically relevant differences those that are greater than 20 ms. Note that all values are between ±2SD from the mean, and more than 70% of values are within ±1SD from the mean. VRT, variability of reaction time.

Bland–Altman plot for values of VRT for first and second tests are shown in Figure 6. Values were found to be distributed around the mean value for the differences, and inside the 95% confidence interval. Considering the clinical criteria (Δ = 20 ms), more than 70% of the differences between measurements were not outside the clinically pre-defined relevant limits. Similarly to the analysis comparing the two modalities (face-to-face vs. online), these findings also strengthened the agreement for VRT between test and retest measurements on the same subject.

**Figure 7:**
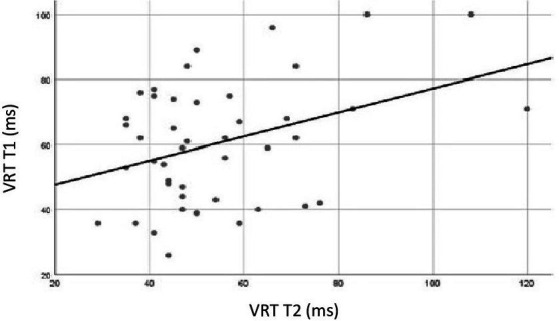
Pearson correlation analysis for variability of correct reaction time (VRT) of Brazilians participants tested two times, test vs. retest. In the *X*-axis, VRT data for participants exposed to retest. In the *Y*-axis VRT data for participants exposed to the first test. Correlation between test and retest was statistically significant for VRT (*r* = 0.37, *p* = 0.01).

Pearson correlation coefficient reached statistical significance for the VRT variable, as shown in Figure 7 (*r* = 0.37, *p* = 0.01). For the other variables, the coefficients did not reach significance.

### 3.4. Brazilians vs. Americans (paired-matched groups)

We performed the statistical analysis separately for online and face-to-face tests. Paired *t*-tests, for robustness check, showed that Americans and Brazilians did not differ in OE, CE, and VRT (Table 7). A small significant difference was found for the RT variable.

**Table 7:** Mean comparisons between Americans and Brazilians, matched by age and sex, and grouped by test modality (face-to-face vs. online).

1: CVAT results for Americans and Brazilians, matched by age and sex (face-to-face).					
	Americans	SD	Brazilians	SD	*p* value
CE	3.93	2.96	3.73	2.13	0.86
OE	0.53	1.06	0.60	0.99	0.87
RT	367.27	33.80	337.67	28.60	0.02
VRT	63.53	21.11	65.33	19.82	0.73
					
2: CVAT results for Americans and Brazilians, matched by age and sex (online)					
	Americans	SD	Brazilians	SD	*p* value
CE	4.40	2.76	4.50	1.90	0.90
OE	0.60	1.07	0.10	0.32	0.21
RT	332.20	28.15	369.60	18.87	0.00
VRT	65.70	17.76	73.00	17.65	0.21
					

SD, standard deviation; CE, commission errors (in units); OE, omission errors (in units); RT, correct reaction time (in ms); and VRT, variability of correct reaction time (in ms).

Regarding agreement for online testing, ICC was statistically significant for VRT (ICC = 0.70, df = 9, *p* = 0.04). In the face-to-face modality, ICC was statistically significant for VRT (ICC = 0.69, df = 14, *p* = 0.02). On both modalities, ICC for the other dependent variables of the CVAT did not reach statistical significance.

## 4. Discussion

We did not find differences in the CVAT performance among different individuals tested online or face-to-face (between-subjects design). Similarly, in the within-subjects design, there were no differences when the same individuals were tested on the two different modalities (online vs. in face-to-face) or across time. Significant agreements were found for VRT comparing modalities, test and retest, and the subjects from the two different countries.

We did not find differences between online and face-to-face modalities in the CVAT performance using different individuals. The between-subjects design allowed us to analyze the effect of the type of administration (modality) independent of any potential learning bias effect.

Regarding the discrepancies observed in the distribution of the variable OE in the robust data analysis, this could be explained based on the fact that the distribution of OE was left skewed. Moreover, the observed differences in OE between modalities (online vs. face-to-face) were too small to be considered clinically relevant.

To account for the intrinsic differences in performance between individuals, we tested the same subject, online and face-to-face. ICC for VRT reached significance. VRT is considered the best predictor of attention deficits in Alzheimer’s disease (Munro Cullum et al., 2014; Schmidt et al., 2020), ADHD (Schmidt et al., 2019), Mild Cognitive Impairment (Schmidt et al., 2020), Chronic Pain (Schmidt et al., 2022), and post-COVID patients who presented early gastrointestinal symptoms (Schmidt et al., 2023). Therefore, our result shows that we can reliably measure the VRT variable independent of the way the test is administered. Considering the importance of the VRT variable in cognitive assessment, this finding reinforces the clinical utility of the CVAT.

On average, no differences were observed between the first and the second tests, with good reliability between test and retest. Moreover, agreement was found for the VRT upon retesting, as shown by the ICC. However, ICC was not significant for the other variables of the CVAT. In the scenario of test and retesting, it is possible that individuals tested across time adapt their response to improve their performance at the second test compared to the first. Subjects tested more than once tend to adapt their way of executing the test: some participants perform faster, with consequently lower RT and higher errors while others perform slower, with fewer errors. In both situations, our results indicate that the VRT remained constant, irrespectively of changes in RT or accuracy. This result is supported by previous studies (White et al., 2019) and emphasizes the role the VRT as reliable measure of attention upon retesting.

It is important to notice that we find a high percentage of agreement for RT. However, the value of *p* for kappa coefficient was not statistically significant. This finding can be explained considering that our sample size included only healthy participants, with most test results within the normal range of values. This statistical phenomenon is, sometimes, referred in the literature as the “kappa paradox” (Zec et al., 2017).

We did not find any significant differences between Brazilian and American participants grouped by testing modality (online or face-to-face), except for a small difference in the RT variable. This small difference was not clinically relevant. The literature shows controversial results on comparisons on cognitive testing between individuals from different cultural backgrounds (Ardila, 2005). However, these studies did not focus on the attention subdomains (Nielsen et al., 2012).

The analyses of the intraclass correlations between Brazilians and American participants matched by sex, age, and level of education indicated that the VRT variable of the CVAT reached significant agreements, independent of the nationality of the subject. This reinforces the stability of the VRT parameter of the CVAT.

### 4.1. Limitations

(1) Our results were obtained in a highly educated sample, since participants were recruited from a university environment, in the case of American subjects, or among medical students, in the case of Brazilian individuals. Further studies should be performed in more diverse populations, at least regarding the level of education; (2) here, the effect size for power analysis was specified to be the minimum meaningful effect, based on the clinical experience of the test (Bakker et al., 2019). However, commonly used interpretation is to refer to effect sizes as small (*d* = 0.2), medium (*d* = 0.5), and large (*d* = 0.8). Therefore, we will need a larger sample for study a small or medium effect; (3) the small sample sizes in the paired comparisons between Americans and Brazilians limit the interpretation of the comparisons. Further research should be conducted in larger samples; (4) one could argue against our study design to evaluate the absence of learning effect on the CVAT, in our third aim, since retest was not necessarily performed using the same modality. Future research should be conducted retesting the same individuals with the same modality (5) a perfectly balanced within-design for the two populations would simplify the structure of the analysis. However, some of the test could not be extended further due to the COVID-19 pandemics. Further studies should be conducted using a perfectly balanced within-design.

### 4.2. Conclusion

There were no differences between online and face-to-face modalities, either when different individuals or the same individuals were tested on the two modalities. We did not detect a learning effect upon retest. Agreement was always found for the sustained attention subdomain (VRT). Moreover, no differences were observed on the CVAT across different cultural boundaries, with VRT remaining the most stable variable.

## Data availability statement

The raw data supporting the conclusions of this article will be made available by the authors, without undue reservation.

## Ethics statement

The studies involving human participants were reviewed and approved by Gafree e Guinle University Hospital. The patients/participants provided their written informed consent to participate in this study.

## Author contributions

DN and SS contributed to the conception, through data collection, analysis, and writing and submission of the manuscript. All authors contributed to the article and approved the submitted version.

## Conflict of interest

The authors declare that the research was conducted in the absence of any commercial or financial relationships that could be construed as a potential conflict of interest.

## Publisher’s note

All claims expressed in this article are solely those of the authors and do not necessarily represent those of their affiliated organizations, or those of the publisher, the editors and the reviewers. Any product that may be evaluated in this article, or claim that may be made by its manufacturer, is not guaranteed or endorsed by the publisher.
